# Possible Mechanisms of Lymphoma Development in Sjögren’s Syndrome

**DOI:** 10.2174/1573395511309010003

**Published:** 2013-02

**Authors:** Lingli Dong, Yu Chen, Yasufumi Masaki, Toshiro Okazaki, Hisanori Umehara

**Affiliations:** 1 Department of Hematology and Immunology, Tongji Hospital, Huazhong University of Science and Technology, Wuhan, Hubei, 430030, China; 2 Division of Hematology and Immunology, Department of Internal Medicine, Kanazawa Medical University, Kahoku-gun, Ishikawa 920-0293, Japan

**Keywords:** Incidence of lymphoma development, lymphoma, lymphoproliferative disease, Sjögren’s syndrome, therapeutic progression.

## Abstract

Primary Sjögren’s syndrome (pSS) is a systemic as well as an organ-specific autoimmune disease characterized by lymphocytic infiltration of the glandular epithelial tissue. SS patients have been reported to be at highest risk of developing lymphoproliferative neoplasms, when compared with patients with other rheumatoid diseases. Factors such as cytokine stimulation, environmental factors, viral infection and genetic events as well as vitamin deficiency may contribute to the development of lymphoma. Over the past few decades, numerous efforts have been made to assess the relationship between lymphoma and SS. These include epidemiological surveys, molecular biologic assessments of clonality and well-linked register cohort studies evaluating the predictive value of clinical, laboratory and histological findings. Nevertheless, the mechanisms and factors predictive of lymphoma development in pSS patients remain to be defined. This review summarizes updated knowledge on the incidence of and risk factors for lymphoma development in pSS patients, as well as discussing the most recent findings on the development and treatment of lymphoma in pSS patients and the possible mechanism of lymphoma development.

## INTRODUCTION

Sjögren’s syndrome is a chronic autoimmune disease characterized by destructive mononuclear cell infiltration of exocrine glands, notably the lacrimal and salivary glands, resulting in dry eyes and dry mouth. During disease progression, Sjögren’s syndrome may extend from an autoimmune exocrinopathy to a systemic disorder including the involvement of non-visceral (skin, joints, muscle, and central and peripheral nervous systems) and visceral (lungs, heart, kidneys, and gastrointestinal and endocrine systems) organs. SS may occur alone (primary SS; pSS) or in association with another autoimmune rheumatic diseases, including rheumatoid arthritis (RA), systemic lupus erythematosus (SLE) and scleroderma, defined as secondary SS. SS is the second most common autoimmune rheumatic disease after RA, with a prevalence of 0.5% in the general population [1]. Although all ages can be affected, it generally tends to occur in menopausal women in the fourth and fifth decades of life. The female: male ratio of SS patients is nine to one [2].

The underlying cause of SS has been an area of extensive investigation, particular over the past two decades, while the pathogenesis remains obscure. In general, SS is thought to be triggered by genetic factors, including the IRF5 and STAT4 genes, as well as variants in the EBF1, FAM167A-BLK and TNFSF4 CHRM3 genes [3-7]; by environmental factors; and by hormonal factors, including estrogen decline, imbalances in the estrogen:androgen ratio [8] and 'X chromosome dosage' [9], factors that ultimately induce immune dysregulation and loss of tolerance.

**Fig. (1):**
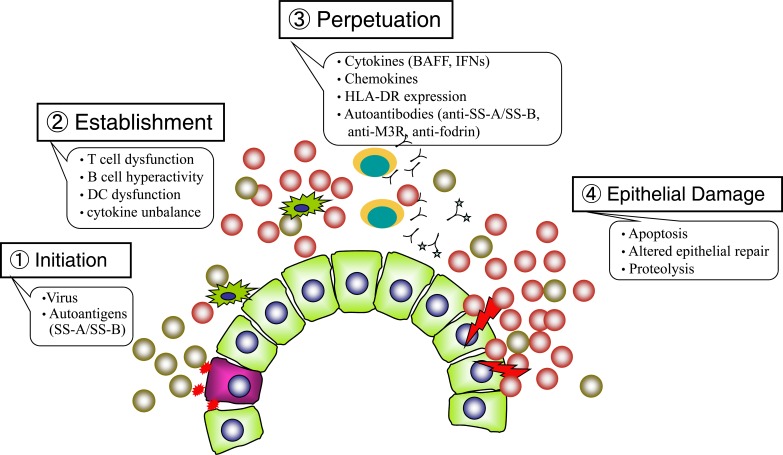
**Pathogenesis of primary SS.** The pathogenesis of SS is multifactorial and includes several different steps. Initiation: An initial event (either viral or non-viral) induces cellular necrosis or apoptosis, subsequently expression of the SS-A and/or SS-B antigens on the glandular-cell surface. Establishment: T cell dysfunction, B cell hyperactivity and abnormal function of dendritic cell contribute to establishment of the histopathological lesions. Perpetuation: Production of cytokines (BAFF, IFNs, etc.) and chemokines by the injured gland promote the migration of lymphocytes and dendritic cells in the gland. IFNs up-regulate cell surface expression of HLA class I or II, and of costimulatory molecules such as CD40L and B-7. Antibodies to SS-A/SS-B antigens are produced by HLA-DR-positive B lymphocytes under the influence of a T-helper lymphocytes. Epithelial damage: Production of IFNs by the dendritic cells perpetuates the process of lymphocyte homing, activation and apoptosis of glandular cells. Thus, this vicious cycle that links the innate and acquired immune systems may occur in SS patients.

The pathogenesis of SS is multifactorial and includes several different steps. The first step is initiation, consisting of an initial signal, either viral or non-viral, to the gland, leading to cellular necrosis or apoptosis, with subsequent expression of the Ro/SSA and/or La/SSB proteins on the glandular-cell surface. The lipid rafts on B cells are altered in primary SS, prolonging the translocation of the BCR into these lipid rafts, and resulting in inappropriately enhanced signaling [10]. The second step in SS pathogenesis is establishment, characterized by persistent abnormal immune responses, including T cell activation, autoantibody production by B cells, dysfunction of dendritic cells (DC) in the salivary gland, the formation of ectopic lymphoid microstructures (i.e. germinal center-like structures) in non-lymphoid organs [11], and dynamic balance between cytokine networks produced by the injured gland [12], all of which contribute to the establishment of the histopathological lesions. The third step in SS pathogenesis is perpetuation, in which secreted cytokines up-regulate the expression of chemokines and cell adhesion molecules on the high endothelial venules of the gland. This process promotes the migration of lymphocytes and DCs into the gland, as well as the secretion of cytokines such as interleukin (IL)-1, IL-6, tumor necrosis factor (TNF)-α, B-cell-activating factor (BAFF) and interferon (IFN)-α by antigen presenting cells (APC). BAFF strongly influences the development of SS in both animal models and patients [13, 14]. IFN-α is produced during viral infections and acts as a potent danger signal that up-regulates cell surface expression of HLA class I and II and costimulatory molecules such as CD40L and B-7 [15]. Antibodies to Ro/SSA antigen (a ribonucleoprotein particle composed of hY-RNAs and 60kDa and 52kDa proteins) are produced by HLA-DR-positive B lymphocytes under the influence of T-helper lymphocytes thought to be involved in the quality control of transcripts synthesized by RNA polymerase III. SSB is an RNA polymerase cofactor that binds viral RNA, suggesting that immunocomplexes containing anti-SSA/anti-SSB antibodies and ribonucleoprotein may stimulate Toll-like receptors (TLRs) by complexing with double-stranded RNA [16]. The autoimmune response is perpetuated and amplified through molecular spreading against the same or other nearby autoantigens [17]. Muscarinic type 3 receptors (M3R) stimulate autoantigen-reactive T cells, resulting in the subsequent production of suppress intracellular Ca2+ influx and inhibit saliva secretion, which are thought to play crucial roles in autoimmune responses [18, 19]. The fourth step in SS pathogenesis is epithelial damage. IFNs produced by dendritic cells further perpetuate lymphocyte homing and the activation and apoptosis of glandular cells. Affected organs in patients with SS have been reported to produce increased IFN-α and to carry a molecular signature characterized by the expression of IFN genes and IFN-dependent transcripts [20]. The progression of self-protein proteolysis and tissue destruction may involve subsequent increases in apoptotic protease activities, together with the abnormal surface exposure of cytoplasmic autoantigens Thus, this vicious cycle linking the innate and acquired immune systems may occur in patients with SS [21] (Fig. **1**).

## INCIDENCE OF LYMPHOMA DEVELOPMENT IN pSS PATIENTS

The most frightening complication of pSS is lymphoproliferative disease (LPD), such as B cell lymphoma. Patients with SS are at higher risk of developing lymphoproliferative neoplasms than patients with other autoimmune disorders, such as SLE, which is associated with medium risk and RA, with lower or even no risk [22, 23]. The high incidence of lymphoma in SS patients was first reported in 1963 [24], and the prevalence of patients with SS who developed lymphoma is approximately 5% [25]. Several recent large cohort studies and a meta-analysis have estimated the lymphoma risk in patients with pSS. One cohort study that included 507 incident patients with pSS, showed that the risk of developing lymphoma was about 16-fold higher in patients who did than did not fulfill the diagnostic American-European Consensus Criteria (AECC) [26]. The risk of lymphoma development in patients who met these criteria increased as follow-up time increased (>10 years). Similar results were observed in a more recent cohort study, which estimated that the relative risk of developing lymphoma was about 16-fold higher in SS patients than in the general population and that this risk increased over time and remained high, even 15 years after pSS diagnosis [27]. The latency periods between the onset of the systemic autoimmune diseases SS, SLE, and RA, and the time of lymphoma diagnosis were 65, 75, and 113 months, respectively [28]. The overall 10-year survival rates were estimated to be 91% for patients with pSS and 69% for patients who developed lymphoma [28]. Moreover, some studies have reported that pSS patients who developed lymphoma were at higher risk of developing a second malignancy [29, 30]. The potential link between lymphoma development and the subsequent development of additional cancers suggests that these cancers may have a common etiology. Alternatively, lymphomas in pSS patients may arise due to suppressed immunity [31], making these patients more susceptible to second malignancies.

Various lymphoma subtypes have been reported associated with pSS. A recent population-based case-control study found that marginal zone lymphoma was most strongly associated with SS, followed by diffuse large B-cell lymphoma (DLBCL), and that these associations remained significant when the 5-year period prior to diagnosis was excluded [32]. This prevalence of lymphoma subtypes is in accordance with previous findings [22-24, 29, 33, 34]. A pooled analysis found that SS was associated with a 6.5-fold increased risk of developing NHL, a 1,000-fold increased risk of developing parotid gland marginal zone lymphoma and increased risks of DLBCL and follicular lymphomas [22]. In contrast, several clinical analyses indicated that MALT and DLBCL lymphomas occurred at a similar frequency [27]. Although SS and NHL subtypes may have common mechanisms of lymphomagenesis, these mechanisms remain unclear. Other histological subtypes of malignant lymphoma have been associated with pSS, including lymphoplasmacytoid [35] and angioimmuoblastic [36] T cell lymphomas. The salivary glands are the most common site of lymphoma development, but extra-nodal sites are also involved, including the stomach, nasopharynx, skin, liver, kidneys, lungs, lymph nodes and bone marrow [37]. No major study to date has assessed gender differences in lymphoma development due to the heavy predominance of female patients with SS.

The Inter Lymph consortium of NHL case-control studies found that patients with secondary SS were at higher risk of NHL development than patients with primary SS, with similar relative risks for NHL subtypes [22]. This observation supports the hypothesis that disease severity, chronic B-cell activation and inflammation may contribute to lymphomagenesis in SS patients [26].

## PREDICTIVE RISK FACTORS FOR LYMPHOMA DEVELOPMENT

Many factors have been reported to predict lymphoma development in SS patients, including lymphadenopathy, swollen salivary glands, probable purpura or skin vasculitis, splenomegaly, glomerulonephritis [34, 38], peripheral involvement (i.e. peripheral neuropathy, such as trigeminal neuropathy, sensorineural deafness, mononeuritis multiplex and small fiber sensory neuropathy), leg ulcers, low grade fever, use of cytotoxic drugs, and younger onset pSS. Laboratory predictors of lymphoma development include anemia, lymphopenia, neutropenia [34], low concentrations of complement factor C3 and/or C4, high serum β-2 microglobulin concentrations, low serum IgM concentrations, the disappearance of a previously positive RF antibodies (RFs) [39], and cryoglobulinemia, especially mixed monoclonal cryoglobulinemia (MMC) [40]. In addition, the particular monoclonal anti-idiotypic antibodies (SF 18/2) against monoclonal RFs were markedly related to the monoclonal proliferation of RF anti-idiotypic positive B cells in patients with SS [41]. The monoclonal rheumatoid factor (RF) cross-reactive idiotypes 17109 and G6 have also been correlated with lymphoma development [40]. The presence of palpable purpura and low C4 concentrations at the first visit were reported to distinguish high-risk patients (type I pSS) from patients with an uncomplicated disease course (type 2 pSS) [38]. Low concentrations of complement factors C3 and/or C4 may increase the risk of unfavorable mutations, resulting in malignancy. CD27 expression has been observed in almost all types of B cell lymphoma, suggesting that this marker may be an early indicator of lymphoma development in pSS patients [42]. The associations between immunosuppressive treatment and lymphoma risk in SS patients are unclear [27, 43]. Cryoglobulinemia, neutropenia, low C4, lymphadenopathy, and splenomegaly are regarded as independent predictors of the development of marginal zone B cell lymphoma (MZBCL), whereas lymphocytopenia was considered as a risk factor for DLBCL development [34].

Recent systemic assessments and clinical analyses have found that CD4+T lymphocytopenia is a strong risk factor for lymphoma development in SS, with the strongest predictor being a lowered CD4+/CD8+ T cell ratio [26, 44]. Traffic of CD4+ cells from the periphery into the tissues may results in a decreased CD4+/CD8 ratio. The detection of germinal center (GC)-like structures in pSS salivary biopsies by light microscopy was recently found to predict NHL development [45], as has the over-production of Fms-like tyrosine kinase3 ligand [46]. Since infiltration by IL-12 expressing cells correlated inversely with infiltration by IL-18 expressing cells, assays for these cytokines may act as histopathological indicators of lymphoma genesis risk in patients with pSS [47]. Additional risk factors related to the development of B cell malignancies in patients with SS may be revealed in future, along with the mechanisms underlying these risk factors. These findings may enable the identification of pSS patients at substantial risk of lymphoma development.

## POSSIBLE MECHANISM OF LYMPHOMA DEVELOPMENT (FIG. 2)

Primary SS has been described as an autoimmune exocrinopathy or epitheliitis, characterized by the infiltration of lymphocytes, primarily T cells (mostly CD4+ cells) and B cells, into glandular epithelial tissue. SS patients with insufficient infiltration for biopsy criterion may be less likely to develop lymphoma, suggesting the importance of interactions among T cells, B cells and epithelial cells [48]. Here we will discuss the recently proposed possible mechanisms underlying the development of B cell lymphomas in these patients, including defects in the apoptosis and mutagenicity of B cells, T cell modulation, persistent antigenic stimulation and the effects of various molecules such as BAFF (BlyS) [13] and type 1 interferons [43].

## T CELL MODULATION/DYSREGULATION

The lymphocytes that infiltrate the minor salivary glands are predominantly T-cells with a bias towards CD4+ rather than CD8+ T cells (ratio >2) [49]. Most CD4+ T cells are of a primed memory phenotype (CD45RO+) and over 50% of all T cells express CD40/CD40L [49]. In SS, T cells can stimulate B cells through the CD40-CD40L interaction in conjunction with the action of various cytokines and chemokines [50], or through the production of BAFF or other promoters of B cell proliferation, which in turn may enhance the tendency toward lymphoma development. In addition, regulatory T cells (Treg) may inhibit protective polyclonal T cell lymphocytic infiltration into mucosal and exocrine tissues, allowing clonal B lymphoid cells to escape immunological surveillance and elimination [51]. Recently, T follicular helper (Tfh) cells were shown to be significant regulators and to participate in the pathogenic processes of autoimmunity [52]. Over-representation of Tfh, with subsequent expression of molecules such as CXCR5, PD-1, ICOS, and CD40L and the secretion of IL-21 in the absence of Blimp-1 [53], were closely associated with B cell activation, differentiation and proliferation [52]. Regulatory B cells (Bregs) are responsible for the proliferation and differentiation of T cells, and the dysregulation of Bregs leads to abrogation of immune tolerance [54].

## ABNORMAL B CELL BIOLOGY (DISTRIBUTION, MUTAGENESIS AND CLONAL EXPANSION)

SS is regarded as a B lymphocyte induced autoimmune disease involving antibody-dependent and -independent mechanisms [55]. Analyses of the distribution of B cell subpopulations from patients with pSS revealed that the number of circulating CD 27+ memory B cells was reduced, while the number in inflamed salivary glands was increased. Remarkably, only SS patients with lymphoma showed an increase in CD27-expressing B cells, including CD27 (high) plasma blasts [56]. Extensive analyses of mature B-cell subsets (Bml-Bm5 classification of peripheral blood B cells in pSS) showed disturbed distributions in patients with pSS, including increases in Bm2/Bm20 and reductions in early Bm5 and Bm5 cells [57], as well as autoimmune abnormalities, when compared with patients with RA or SLE or normal subjects. These disturbances may reflect alterations in B cell trafficking and/or differentiation, which may contribute to lymphoma development [58].

Recent studies have focused on the role in pSS pathogenesis of BAFF, a member of the TNF super family, also called the B lymphocyte stimulator (BLys), and the proliferation inducing ligand APRIL, which participates in B-cell activation. The BAFF/APRIL system regulates B-cell survival, differentiation and proliferation [59]. Both BAFF and APRIL are over expressed in pSS patients [60]. The over expression of BAFF in infiltrated salivary glands may contribute to B cell survival, aggregation, altered differentiation and tolerance, as well as in lymphoma development.

Lymphoma development in SS may also involve B cell mutagenicity [43, 61]. The proportion of B cells expressing mutated V(H) genes was significantly higher in B-cells isolated from parotid gland than in circulating B cells. Furthermore, V(H) gene analysis of B cells isolated from salivary glands revealed biased usage of the V(L) chain gene [62]. And this aberrant mutation patterns may results from the ectopic microenviromental influences to which the GC-like structures are exposed [63]. Immunoglobulin generation occurs early during B cell development in the bone marrow. During later steps of B cell development, immunoglobulin undergo recombination, somatic mutation and isotype switching, events that require breaking and reconnecting DNA, increasing the risk of chromosome translocation of oncogenes, such as Bcl-2 and c-Myc, to immunoglobulin loci on chromosome 14q32 [64]. Mutagenesis during these processes may result in the generation of B cells with defective apoptosis and enhanced proliferation, favoring lymphoma development [43]. Chronic B cell activation in SS salivary glands, especially in the GC-like structures, has been found to induce activation cytidine deaminase (AID), which plays an important role in somatic hyper mutation and immunoglobulin of Ig genes during the affinity maturation of B cells, and to creates a risk for proto-oncogene formation [25, 65]. BAFF was shown to up regulate AID, promoting class switch recombination in human B-lymphocytes. Since JAK2 kinase has been shown essential to the proliferation of hematopoietic stem cells, JAK2 mutations may be critical in the pathogenesis of myeloproliferative disease [66]. A significant percentage of SS patients who develop lymphoma have Fas mutations, suggesting that the somatic disruption of Fas may play a role in lymphoproliferation [67].

The transition from an autoimmune state to lymphoma is a multi-step process, with chronic stimulation by an exoantigen or an autoantigen driving the proliferation of specific B cells and increasing the frequency of their transformation, thus contributing to tumor development [42, 60, 68]. In addition, immunoglobulin is important in stimulating RF-producing clones and in the development and expansion of myoepithelial sialadenitis (MESA)-associated clones. MALT lymphomas express a unique antibody repertoire (i.e. the 17.109 idiotype) along with reactivity to RF, which is encoded by Hum kv325 [69]. In addition, marginal B lymphocytes express hyper-mutated immunoglobulins with preferential selection of Igs with RF properties which may have a role in T cell co-stimulation [25]. Prolonged inflammation may be due to the persistence of antigenic stimulatory organisms such as Helicobacter pylori [70]. Helicobacter pylori together with T cells has been reported to stimulate the growth of gastric MALT lymphomas, with antibiotics leading to MALT lymphoma regression [71]. Thus, chronic antigenic stimulation by Helicobacter pylori may play a role in the development of MALT-type B cell lymphoma [72]. Helicobacter pylori may induce the production of large amounts of IgG in the salivary glands, lead to uncontrolled RF-producing B cell clones in the ectopic germinal centers and vigorous expansion, making their DNA susceptible to mutational events [73].

## CYTOKINES

Cytokines, as regulators of the immune system, contribute to both the pathogenesis of SS and to lymphoma development in these patients [48]. Th1 and Th2 cytokine profiles have been studied in the blood and salivary gland tissues of patients with SS [58], with both types expressed by lymphocytes infiltrating their salivary glands. Th-2 cytokines may be predominant during the early phase of SS, with a shift towards Th-1 cytokines associated with advanced lymphocytic infiltration during later stages of the disease [74]. This shift may be controlled by B-cells as immunopathological lesions progress [75]. BAFF concentrations are markedly increased in the serum and target organs of patients with SS. Increased BAFF production may result in continuous B cell activation, lead to B cell lymphoma development [13]. Type I interferons can increase BAFF expression, resulting in the development of B cell lymphomas [43].

Levels of Fms-like tyrosine kinase 3 ligand (Flt-3L) were shown to be closely related to SS disease severity and lymphoma development [46]. IL-6 may play be involved in the rearrangements of immunoglobulin V region genes during B-cell proliferation [76]. The chemokines CXCL13 and CCL21 were expressed only in areas of benign lymphoepithelial lesions, especially in tissues with lymphomatous proliferation, whereas CXCL12 was expressed selectively in areas of malignant lymphocytic infiltration, mainly in ductal epithelial cells. These findings suggest that CXCL12 is a primary chemokine associated with malignant B cell regulation [77]. The complicated interactions and expression of various factors in the SS microenvironment may be indispensable in lymphomagenesis.

## VIRUSES

Several viruses may be involved in the pathogenesis of pSS, including herpes virus 6, cytomegalovirus, Epstein-Barr virus, human T lymphotropic virus type I, human immunodeficiency viruses, human intracisternal A-type retroviral particle, human retrovirus 5, and coxsackie virus 6. In addition, the microorganism chlamydia psittaci may be involved in pSS pathogenesis. These viruses may not only initiate the immune response [78] but may act as sustaining antigens, causing chronic stimulation and persistent B cell proliferation. One study, however, found no relationship between lymphoma development and infection with EBV, cytomegalovirus, human immunodeficiency virus, or H. pylori [27], suggesting that infection may not play a leading role. Although experimental, epidemiologic, virological and clinical findings have revealed a close association between hepatitis C virus (HCV) infection and SS [79], the association between B cell lymphoma and HCV remains unclear [80, 81]. The etiopathogenic mechanisms of lymphoma development in patients with SS and HCV are similar [82, 83]. Both are characterized by parotid enlargement and vasculitis, the presence of RF and mixed type 2 cryoglobulins, the predominance of MALT lymphomas and an elevated frequency of primary extra-nodal involvement of organs in which HCV replicates (exocrine glands, liver and stomach) [81]. Moreover, patients with HCV-associated SS were reported to have a higher frequency of malignancies than patients with either alone [84]. HCV is a lymphotropic virus that predominantly affects B cells, which may result in clonal proliferation and the induction of B-cell malignancies. Human herpes virus infection has also been linked with the development of MALT lymphoma in SS patients [85].

## ONCOGENE MOLECULES AND OTHERS

Together with persistent antigenic stimulation, additional oncogenic events such as the inactivation of proto-oncogenes are likely required for lymphoma development in SS [86]. The presence of antibodies to p53 in sera from patients with SS and NHL [87] indicated that dysregulation of this tumor suppressor gene may be involved in the developmental mechanism of low grade MALT lymphoma, whereas complete loss of p53 function was related to high grade transformation [88]. A high frequency of t(14,18) translocations have been observed in salivary gland lymphomas from SS patients [89]. Moreover, the frequency of the classical t(11;18) (q21;q21) translocation was much lower in SS patients with extra gastrointestinal than with gastric MALT lymphomas [90]. Evidence of defective repair of a pro-mutagenic DNA base lesion, O6-methylguanine, has been observed in the lymphocytes of patients with pSS predisposed to lymphoma [91], suggesting that these patients may have defective DNA repair mechanisms. Other genetic events, such as bcl-2 or c-Myc amplification, trisomy 3 or 18, may facilitate the progression of low-grade MALT lymphoma to a more malignant high-grade lymphoma [43, 48, 64, 91, 92]. Activation of the enzyme cytidine deaminase (AID) has been associated with somatic hypermutation and class switch recombination [65].

Most recently, genetic variations in complement C3 and germinal and somatic abnormalities of the A20 (TNFAIP3) gene [93, 94], as well as vitamin-D deficiencies [95], have been associated with lymphoma in patients with pSS. Since vitamin B12 deficiency can cause pernicious anemia, it may also play a role in accelerating the development of lymphoma, especially multiple myeloma [32].

Taken together, the complicated interactions and factors in the microenvironment of SS play an indispensable role in the genesis of lymphoma in patients with this autoimmune disease.

## CLONALITY ANALYSIS IN LYMPHOMA DEVELOPMENT

In contrast to the focal sialadenitis of the minor salivary glands frequently observed in pSS patients, lymphocytes infiltrating the major salivary glands often form secondary lymph follicles. B cells have been shown to infiltrate the glandular duct epithelium in pSS patients, thereby contributing to the characteristic pattern of chronic lymphocytic inflammation, so called MESA or benign lymphoepithelial lesion. Patients with clonally expanded B cells in their salivary glands may be at high risk of developing lymphomas, and chronic exogenous or endogenous stimulation in MESA has been hypothesized to play an important role in the lymphoproliferative process during the course of SS, through the restricted usage of immunoglobulin heavy chain complementarities determining region 3 (IgVH-CDR3) from MESA-associated clones [68, 96].

Immunoglobulin heavy chain gene rearrangement has been used diagnostically to determine monoclonality in pSS patients with lymphoma [97]. Using this method, we assessed B cell clonality in lymphoproliferative tissues from 6 patients with pSS and lymphoproliferative disorders or lymphoma. Three longitudinally observed patients showed progressive clonal expansion with the presence of the same subclone in different tissues during the course of disease, with one developing MALT lymphoma in the parotid gland. The other three SS patients had malignant B cells between nodal sites and salivary glands [98]. Table **1** summarizes findings regarding monoclonality and lymphoma development in 45 patients with SS. Of these 45 patients, 42 showed evidence of monoclonal B cell expansion by PCR, although only 16 developed lymphoma, indicating that the simple detection of B cell clonality by PCR cannot be used as a criterion for diagnosis of B cell lymphoma. The malignant lymphoma clones established from 13 SS patients were found to be derived from the initial clones, since sequence analysis showed that the amino acid sequence motifs in the CDR3 were conserved [68, 96, 99-103]. Although these patients are not representative of all patients with SS, it is likely that patients with the persistent monoclonal B cell expansion in follow-up biopsy specimens are at higher risk of developing lymphoma and that malignant clones are derived from those in initial lymphoproliferative glandular tissues. In contrast, other studies showed that the detection of monoclonality by immunoglobulin gene rearrangement was not a reliable predictor of clinical behavior in MESA, inasmuch as initial and follow-up biopsy specimens from the same patients were identical [96]. Similar findings were observed in our study [98]. Thus, molecular genetic analysis of monoclonality has little practical value in the clinical diagnosis of salivary gland lymphoma in MESA, and these patients should be diligently followed-up for evidence of lymphoma development [104]. Using semi-nested PCR method with FR2/LJHVLJH, FR3/LJH and FR1c/JH1-6 primers, B cell monoclonality in the minor labial salivary gland (MSG) were detected in 87% of SS patients, but in only 19% of control subjects. Since this PCR method is hypersensitive, the presence of B cell monoclonality in MSG may predict malignant clonal expansion [105].

## THERAPEUTIC ADVANCE IN LYMPHOMA ASSOCIATED WITH pSS

Since SS is at the crossroads of autoimmune disease and lymphoma, treatment of SS-associated lymphoma should target both the autoimmune and neoplastic natures of this disease. The most widely used chemotherapy regime is CHOP (cyclophosphamide, adriamycin, vincristine and oral prednisolone), or CVP (cyclophosphamide, vincristine, and prednisone) [106]. Rituximab (RTX), an anti-CD20 monoclonal antibody, which targets B cells and results in the depletion of activated B lymphomas, has been considered as a promising treatment in pSS patients with lymphoma [107]. However, the effectiveness of RTX monotherapy on B cell lymphoproliferation in SS is unclear [108], with SS patients with parotid MALT type being especially resistant. RTX treatment may show good effects only during the early, active stage [109]. while combined the chemotherapy with rituximab (RTX) was well tolerated and effective in all 4 [110] and in 4 of 5 [111] patients with pSS-associated aggressive B cell lymphomas, which became standard therapy for the past 5-10 years [107]. In addition, treatment with 2-chloro-2'-deoxyadenosine, an adeoxyadenosine analog that acts independently on cell division, resulted in complete responses in 3 of 4 patients with SS associated B cell lymphoproliferation during 4 years of follow up. Larger controlled trials are therefore warranted to assess the effectiveness of these two regimens [112].

Inhibition of BAFF, either as monotherapy or combined with RTX, is considered as a promising therapeutic intervention in pSS patients [113]. A monoclonal antibody against (BAFF), belimumab, has recently been licensed for use in patients with SLE [114] and should be assessed in clinical trials in patients with pSS. Moreover, combined therapies with agents directed against interferons and other cytokines, chemokines, adhesion molecules and other cell-cell interactions may have curative effects [115], although large numbers of experimental and clinical studies are needed.

## SUMMARY

SS, at the crossroads of autoimmune disease and lymphoma, is a powerful model for providing potential insight into the pathogenic mechanisms responsible for lymphoma development. Focusing on pre-lymphomatous stages is crucial to better understanding the entire lymphomagenesis process in SS. Monoclonal B cell proliferation in the salivary glands frequent occurs in patients with pSS, but these cells are not necessarily malignant. PCR amplification of immunoglobulin heavy chain gene rearrangements was not a reliable predictor of lymphoma development. DNA sequencing may provide insights into clonal progression, although its ability to predict lymphoma development remains unclear. Clinical symptoms and parameters, such as parotid enlargement, palpable purpura, low C4 levels, and especially CD4+ T lymphocytopenia, are valuable indicators of lymphoma development. Since the association between pSS and lymphoma is real, SS patients should be examined carefully to assess disease progression and identify individuals who are at substantially increased risk of lymphoma development. Optimal interventions against these risks are also needed, although the mechanisms underlying lymphoma development in pSS are complicated and as yet undetermined.

## Figures and Tables

**Fig. (2):**
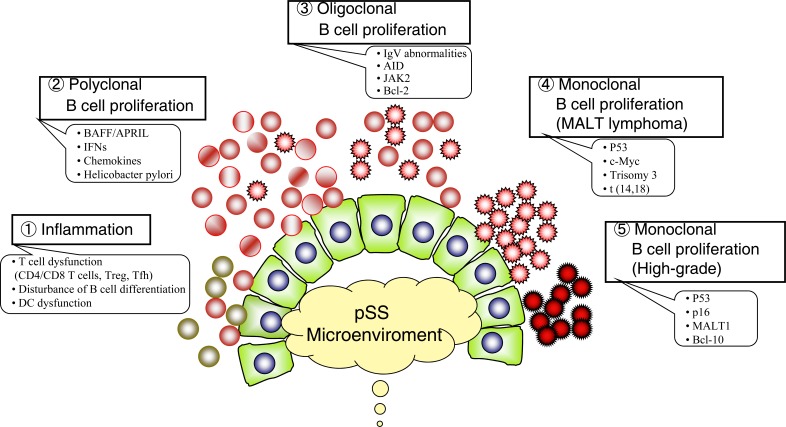
**Hypothetical model of lymphoma development in SS patients.** The transition from autoimmune state to lymphoma is a multi-step process and that chronic stimulation by exoantigen or autoantigen plays an important role in the development of lymphoma by driving the proliferation of specific B cells and increasing the frequency of their transformation. Inflammation: Infiltration of CD4+ T cells, memory B cells and dendritic cells in the minor salivary glands perpetuate chronic inflammation. (Polyclonal B cell proliferation: Increased production of BAFF and IFNs in SS patients cause polyclonal B cell proliferation and thereby contribute to the characteristic pattern of myoepithelial sialadenitis (MESA) or benign lymphoepithelial lesion. Oligoclonal B cell proliferation: BAFF specifically regulates B lymphocyte proliferation and survival, altered B cell differentiation. Chronic stimulation by exoantigen or autoantigen may drive the proliferation of specific B cells through restricted usage of immunoglobulin heavy chain complementarity determining region 3 (IgVH-CDR3) and increasing the frequency of their transformation. Monoclonal B cell proliferation: During B cell development, immunoglobulins undergo recombination, somatic mutation and isotype switching. These events may increase the risk of translocation of oncogenes such as Bcl-2 and c-Myc to immunoglobulin loci (chromosome 14q32). Transformation to high grade malignancy: Defect of P53 tumor-suppressor activity, high frequency of t(14,18) translocation, amplification of bcl-2 and/or c-Myc, and trisomy 3 may facilitate the progression of low-grade MALT lymphoma to more malignant high-grade lymphoma.

**Table 1.** The Literatures of Monoclonality and Lymphoma Development

Author	Year of Publication	Number of Cases	SS➜ Lymphoma	Same Clone†	Different Clone‡
Diss PC [116]	1993	1	1SS➜ MALToma		1
Pablos JL [99]	1994	14	1SS➜ B cell neoplasm	1	
Jordan R [117]	1995	11	4SS➜ lymphoma	4§	
Lasota J [118]	1997	1	1SS➜ MALToma		1
De Vita S [96]	1997	6	1SS➜ Lymphoma (DLB)	1	
Bathler DW [68]	1998	7	5SS➜ lymphoma	4	1
Aiello A [100]	1999	1	1SS➜ MALToma➜FL	1	
Gellrich S [101]	1999	2	0➜ 0	0	0
Gasparotto D [102]	2003	1	1SS➜ Lymphoma (MZB)	1	
Hansen A [103]	2006	1	1SS➜ Lymphoma (MZB)	1	
Total	45	16	13	3	

† Same clone: malignant clone is from the initial lymphoproliferative glandular tissues by PCR and/or sequence, data to identify same monocloloanl or mutated monoclonal B cell expansion with conserved amino acids sequence motifs in their CDR3.

‡ Different clone: different size of monoclonal bands by PCR or same size bands with distinct IgVH-CDR3 between initial lymphoproliferation and lymphoma.

§ Without sequence data, the result is from PCR. DLB,diffuse large B cell lymphoma; FL, follicular lymphoma; MZB,marginal zone B cell lymphoma

## ABBREVIATIONS

SS: = Sjögren’s syndrome
pSS: = Primary Sjögren’s syndrome
SIRs: = Standardized incident rates
SLE: = Systemic lupus erythematosus
MALT: = Mucosa associated lymphoid tissue
MESA: = Myoepithelial sialadenitis
BAFF: = B-cell-activating factor;
